# Fas (CD95) induces rapid, TLR4/IRAK4-dependent release of pro-inflammatory HMGB1 from macrophages

**DOI:** 10.1186/1476-9255-7-30

**Published:** 2010-06-17

**Authors:** Feng Wang, Ziyue Lu, Michael Hawkes, Huan Yang, Kevin C Kain, W Conrad Liles

**Affiliations:** 1 Department of Medicine, Toronto General Research Institute, McLaughlin-Rotman Centre for Global Health, McLaughlin Centre for Molecular Medicine, University Health Network, University of Toronto, Toronto, Ontario, Canada; 2 Laboratory of Biomedical Science, the Feinstein Institute for Medical Research, Manhasset, NY, USA

## Abstract

Although Fas (CD95) is recognized as a death receptor that induces apoptosis, recent studies indicate that the Fas/FasL system can induce pro-inflammatory cytokine production by macrophages independent of conventional caspase-mediated apoptotic signaling. The precise mechanism(s) by which Fas activates macrophage inflammation is unknown. We hypothesized that Fas stimulates rapid release of high mobility group box 1 (HMGB1) that acts in an autocrine and/or paracrine manner to stimulate pro-inflammatory cytokine production via a Toll-like receptor-4 (TLR4)/Interleukin-1 receptor associated kinase-4 (IRAK4)-dependent mechanism. Following Fas activation, HMGB1 was released within 1 hr from viable RAW267.4 cells and primary murine peritoneal macrophages. HMGB1 release was more rapid following Fas activation compared to LPS stimulation. Neutralization of HMGB1 with an inhibitory anti-HMGB1 monoclonal antibody strongly inhibited Fas-induced production of tumor necrosis factor (TNF) and macrophage inflammatory protein-2 (MIP-2). Both Fas-induced HMGB1 release and associated pro-inflammatory cytokine production were significantly decreased from *Tlr4*^*-/- *^and *Irak4*^*-/- *^macrophages, but not *Tlr2*^*-/- *^macrophages. These findings reveal a novel mechanism underlying Fas-mediated pro-inflammatory physiological responses in macrophages. We conclude that Fas activation induces rapid, TLR4/IRAK4-dependent release of HMGB1 that contributes to Fas-mediated pro-inflammatory cytokine production by viable macrophages.

## Introduction

Fas (CD95) is a 48-kDa, type I transmembrane protein member of the TNF receptor family [1]. The endogenous ligand for Fas is FasL (CD178), a 40-kDa, type II homotrimeric transmembrane protein member of the TNF gene family [1,2]. The Fas/FasL (APO-1/APO-1L; CD95/CD95L) system was first reported, and is generally recognized, as a major regulator of the extrinsic pathway of caspase-dependent apoptosis [3-6]. Fas-mediated apoptosis has been extensively studied for its non- or anti-inflammatory role in tissue injury and organ dysfunction [7-9]. However, accumulating evidence indicates that Fas can mediate myeloid differentiation factor 88 (MyD88)/interleukin-1 receptor-associated kinase-4 (IRAK4)-dependent pro-inflammatory responses via mechanisms distinct from its role in apoptotic programmed cell death [10-14].

HMGB1 protein was first described almost 30 years ago as a non-histone chromosomal protein with high electrophoretic mobility [15,16]. As a DNA binding protein, HMGB1 is involved in maintenance of nucleosome structure and regulation of gene transcription [17,18]. In addition to its role in regulation of transcription, HMGB1 has been shown to activate pro-inflammatory responses when released by necrotic cells into the extracellular milieu [19]. HMGB1 has been implicated in the pathogenesis of a number of diseases associated with inflammation and tissue injury, including sepsis [20-23]. Recent evidence indicates that HMGB1 can be released not only from necrotic cells but also from activated macrophages to act as an 'endogenous dangerous signal' or 'alarmin' [24]. Several cognate cell surface receptors have been proposed for HMGB1, including receptor for advanced glycation end products (RAGE), TLR2 and TLR4 [25-27]. HMGB1-induced proinflammatory cytokine production by macrophages has been reported to be TLR4-dependent [28-30].

In this study, we demonstrate that activation of Fas induces rapid release of HMGB1 from a murine macrophage (mϕ) cell line and primary murine peritoneal mϕ that contributes to Fas-induced pro-inflammatory cytokine production. Furthermore, using genetically defined mice, we show that TLR4/IRAK4-dependent mechanisms are involved in Fas-induced HMGB1 release and pro-inflammatory cytokine production by mϕs. These findings identify a novel mechanism for Fas-mediated pro-inflammatory responses and implicate important physiological cross-talk between Fas, TLR4, and HMGB1.

## Materials and methods

### Reagents

Recombinant mouse Fas ligand (CD178) was obtained from R&D system (Minneapolis, MN, USA). Anti-mouse Fas monoclonal antibody (mAb) Jo2 IgG_2 _was obtained from BD Bioscience (Mississauga, ON Canada). Lipopolysaccharide (LPS, *Escherichia coli *0111:B4) was obtained from Sigma (St Louis, MO, USA). Inhibitory anti-HMGB1 neutralizing monoclonal antibody was provided by Dr. Huan Yang from the Feinstein Institute for Medical Research (Manhasset, NY, USA) [30]. Mouse IgG_2b _isotype control (R&D System, MN, USA) was used as a negative control antibody.

### Primary murine peritoneal macrophage isolation and culture

Animal use was performed in compliance with current University of Toronto institutional guidelines, and animal protocols were approved by the Animal Care Committee of the University of Toronto. Peritoneal mϕ were obtained by peritoneal lavage from wild type, *Tlr2*^-/-^, *Tlr4*^-/-^, *Irak4*^*-/- *^mice (all mice were maintained on the C57BL/6 genetic background; age: 8-10 weeks; weight: 20-25 g) and Fas-deficient *Fas*^*lpr *^mice (The Jackson Laboratory, Maine, USA). Briefly, 10 ml of sterile RPMI was injected into the peritoneal cavity, and the contents of the cavity were gently massaged. A similar volume was then removed from the abdomen, and 2 × 10^6 ^cells/well were seeded in a volume of 2 ml/well in a 6-well tissue culture plate.

### Cell culture and treatment

The murine mϕ cell line RAW264.7 (American Type Cell Collection, Rockville, MD, USA) and primary murine peritoneal mϕ were maintained in RPMI 1640 (Sigma; Mississauga, ON, Canada), containing 10% heat-inactivated fetal bovine serum (FBS) (Sigma, Mississauga, ON, Canada) supplemented with gentamicin reagent solution (Invitrogen; Burlington, ON, Canada), at 37°C in 5% CO2-enriched air. Cells (2 × 10^6^/well) were plated in 6-well tissue culture plates at a volume of 2 ml/well in RPMI 1640 containing 10% fetal bovine serum and pretreated with or without inhibitory anti-HMGB1 neutralizing monoclonal antibody (IgG_2b _mAb) (0.25 μg/ml) or a negative isotype (IgG_2b_) control (IgG_2b_) mAb (0.25 μg/ml). Macrophages were stimulated with recombinant mouse Fas ligand (mFasL, 0.25 μg/ml), or activating anti-murine Fas mAb Jo2 (IgG_2_, 0.25 μg/ml), or LPS (0.25 μg/ml). mFasL and mAb Jo2 were pretreated with Detoxi-Del™ Endotoxin Removing Gel (Pierce Biotech: Rockford, IL, USA) to remove any contaminating LPS. Samples of culture supernatant were obtained at multiple time points. Cell count and viability were determined microscopically by 0.4% trypan blue dye exclusion. The concentration of endotoxin in each wells were tested with Prostate LAL (PSD10, MA, USA). In our experiments, clot formed only in the LPS treated group indicating that endotoxin levels in all other groups were less than 0.25 EU/ml.

### Cell viability assay

Cell viability was determined by trypan blue exclusion using a Vi-Cell XR Cell Analyzer (Beckman Coulter, CA, USA).

### Preparation of cellular extracts

Cells were harvested at the designated times and washed twice with cold PBS; nuclear and cytoplasmic extracts were prepared according to the method of Schreiber et al [31]. Briefly, the cell pellets were resuspended in 400 μl cold buffer A (10 mM HEPES, pH 7.9, 10 mM KCl, 0.1 mM EDTA, 0.1 mM EGTA, 1 mM DTT, 0.5 mM PMSF). Cells were allowed to swell on ice for 15 min, after which 25 μl of a 10% solution of Nonidet P-40 was added, and the tube was vortexed vigorously for 10 s. The lysate was centrifuge at 4°C at 15000 g for 3 min, and the nuclear pellet was resuspended in 50 μl ice-cold buffer B (20 mM HEPES, pH 7.9, 0.4 M NaCl, 1 mM EDTA, 1 mM EGTA, 1 mM DTT, 1 mM PMSF). After vigorously rocking at 4°C for 15 min on a shaking platform, the nuclear extract was centrifuged for 5 min at 4°C, and the supernatant was frozen at -80°C. The protein content of the different fractions was determined by Bradford assay.

### Concentration of conditioned cell culture medium

After incubation, cell medium were collected and concentrated 10-fold using a 7 ml ICON concentrator (Pierce Biotechnology, Rockford, IL, USA) according to the manufacturer's protocol. The final protein concentration of cell medium of the negative control group was 5 μg/μl. The concentrated samples were boiled for 5 min and used for detection of HMGB1 by immunoblot analysis.

### HMGB1 immunoblot analysis

Proteins in concentrated culture media and cell extracts were resolved on 10% SDS-PAGE and transferred to a polyvinylidene fluoride membrane. After blocking with 5% non-fat milk at room temperature for 1 hr, the transfer membranes were incubated for 1 hr with primary antibodies specific for HMGB1 (Abcam, MA, USA), proliferating cell nuclear antigen (PCNA; BD Biosciences, CA, USA) and β-actin (Cell Signaling, Pickering, ON, CANADA), respectively. After incubation with peroxidase-conjugated secondary antibodies for 1 hr at 25°C, the signals were visualized by 3'-diaminobenzidine detection (Bio-Rad Laboratories, Hercules, CA, USA) according to the manufacturer's instructions.

### Measurement of cytokine production

The levels of TNF-α and MIP-2 in culture supernatants were measured by cytokine-specific enzyme-linked immunosorbent assay (ELISA) using previously validated DuoSet antibody pairs (R&D Systems, MN, USA) according to the manufacturer's instructions.

### Statistical analysis

All data are presented as mean ± standard deviation (SD). The data were analyzed by the *t*-test (parametric) and by the Mann-Whitney *U *test (non-parametric) for comparisons between two independent groups. A *P *value < 0.05 was considered to be statistically significant.

## Results

### Activation of Fas induces rapid release of HMGB1 from viable mϕ

**Figure 1:**
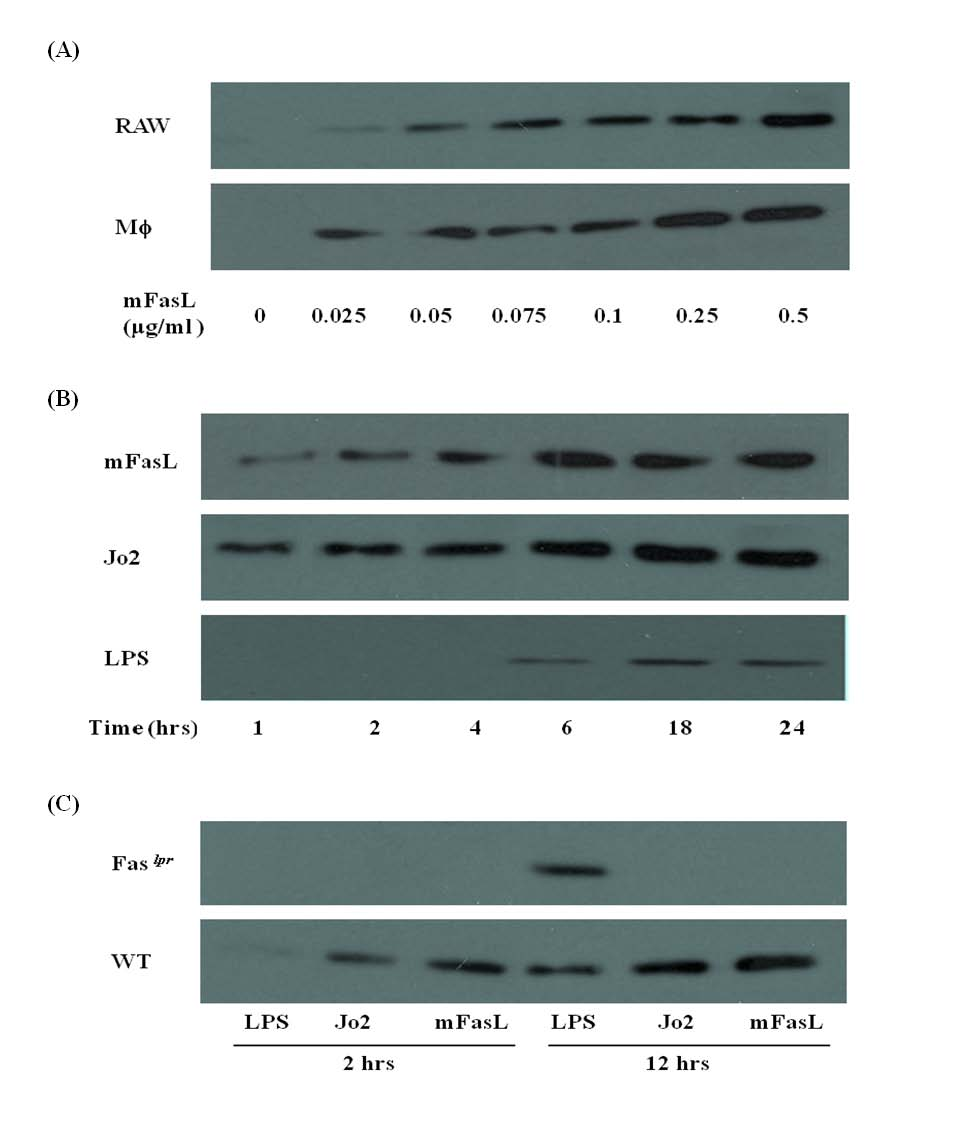
**Fas activation induces rapid release of HMGB1 from primary murine peritoneal mϕ and RAW264.7 cells **. (A) HMGB1 in cell culture-conditioned medium was detected by immunoblot as described in Materials and Methods following treatment with recombinant mFasL (0 - 0.5 μg/ml) for 2 hr. (B) HMGB1 in cell culture-conditioned medium was detected by immunoblot as described in Materials and Methods following treatment with mFasL, mAb Jo2 and LPS respectively (0.25 μg/ml for each stimulus) for the times indicated (0 - 24 hr). (C) HMGB1 in cell culture-conditioned media from primary wild-type and *Fas*^*lpr *^murine peritoneal mϕs culture-conditioned medium was detected by immunoblot as described in Materials and Methods following treatment with mFasL, mAb Jo2 and LPS (0.25 μg/ml for each stimulus), respectively, for the times indicated (0 - 24 hr). All the results are representative of 3 independent experiments that yielded similar results.

Treatment with recombinant mFasL (0 - 0.5 μg/ml) induced release of HMGB1, as detected by immunoblot, from RAW264.7 cells and primary murine peritoneal mϕ in a dose-dependent manner (Figure 1A). Because LPS is recognized to be an important stimulus for activation of HMGB1 release from mϕ [32], the release of HMGB1 was compared at different times (1-24 hr) following incubation with mFasL, activating anti-Fas mAb Jo2 (0.25 μg/ml), and LPS (0.25 μg/ml), respectively (Figure 1B).

In the absence of mFasL, HMGB1 release from wild-type mϕ was not detected at any time. Following stimulation with either mFasL or mAb Jo2, the concentration of HMGB1 released into the medium increased overtime. We observed that the time courses of mFasL and LPS-induced HMGB1 secretion were different. Whereas release of HMGB1 following stimulation could be detected as early as 1 hr following stimulation with mFasL, LPS-induced release of HMGB1 could not be detected until 6 hr (Figure 1B).

To demonstrate conclusively that the release of HMGB1 induced by treatment with either mFasL or mAb Jo2 is Fas-specific, we performed experiments using mϕ from Fas^*lpr *^mice which are functionally Fas-deficient. HMGB1 was detected by immunoblot in the culture supernatant of Fas^*lpr *^mϕ following treatment with LPS. In contrast, HMGB1 release could not be detected from Fas^*lpr *^mϕ stimulated with either mFasL or mAb Jo2 for 12 hr (Figure 1C).

**Figure 2:**
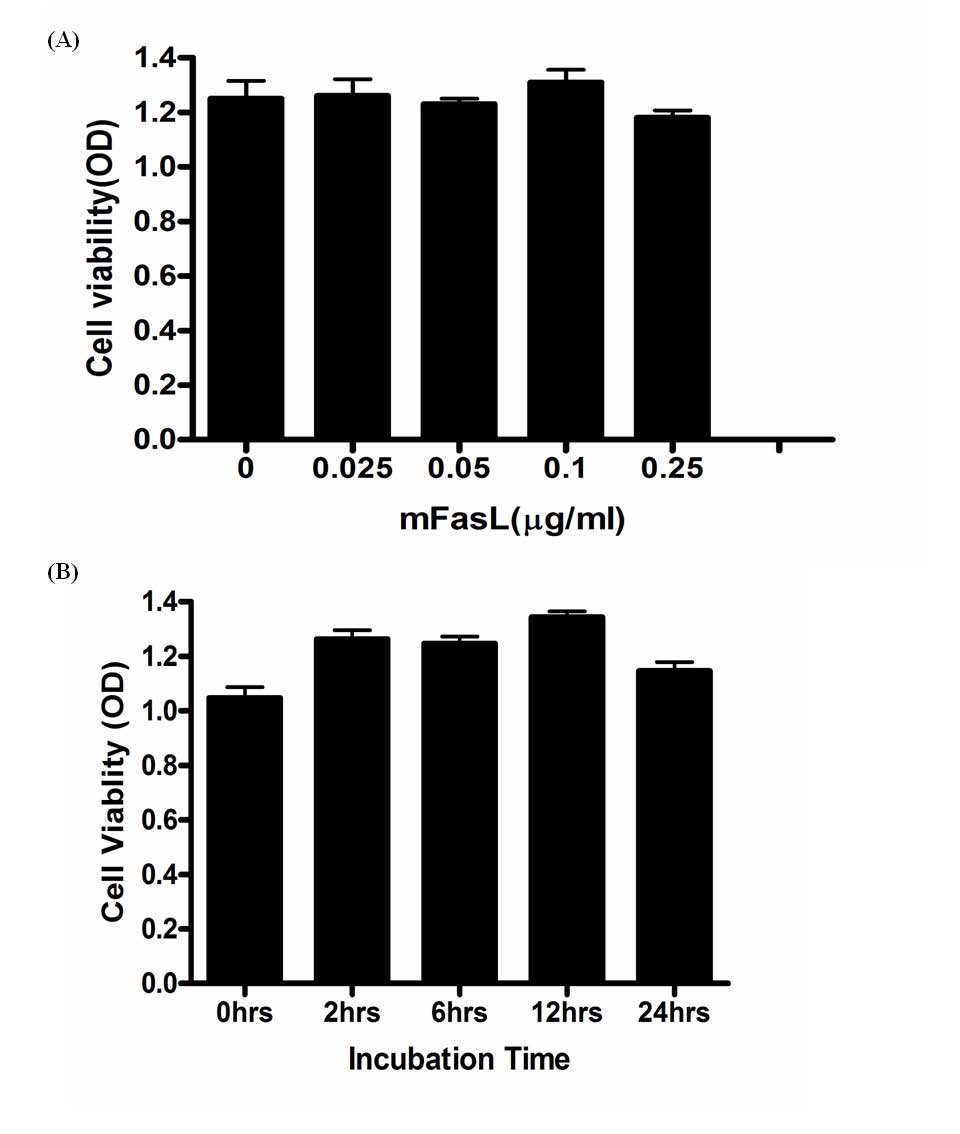
**Fas activation for 24 hr does not affect viability of primary murine peritoneal mϕ**. (A) Dose response analysis of viability of primary murine peritoneal mϕ treated with recombinant mFasL (0-0.5 μg/ml); (B) time course (0 - 24 hr) analysis of primary peritoneal mϕ following treatment with recombinant mFasL (0.5 μg/ml). Cell viability was determined by trypan blue exclusion using a cell viability analyzer as described in Materials and Methods. Results are presented as mean ± SD of 3 independent experiments, n = 3/group. * *P *≤ 0.05; **P ≤ 0.01.

Because HMGB1 can be released not only passively by apoptotic and necrotic cells but also by activated macrophages via a death-independent pathway [33,34], viability of murine peritoneal mϕ was examined after incubation with mFasL at various dosages and time courses. Viability was not significantly affected by incubation with mFasL (0 - 0.5 μg/ml) over a 24 hr time period (Figure 2A, B), consistent with our previous report [14]. Similarly, the viability of RAW 264.7 cells was not affected by overnight incubation with mFasL (0.5 μg/ml) as previously reported [14]. These results indicate that Fas induces HMGB1 release from mϕ via a mechanism that does not involve or depend on cell death.

### Fas induces translocation of HMGB1 to the cytoplasm of mϕ

**Figure 3:**
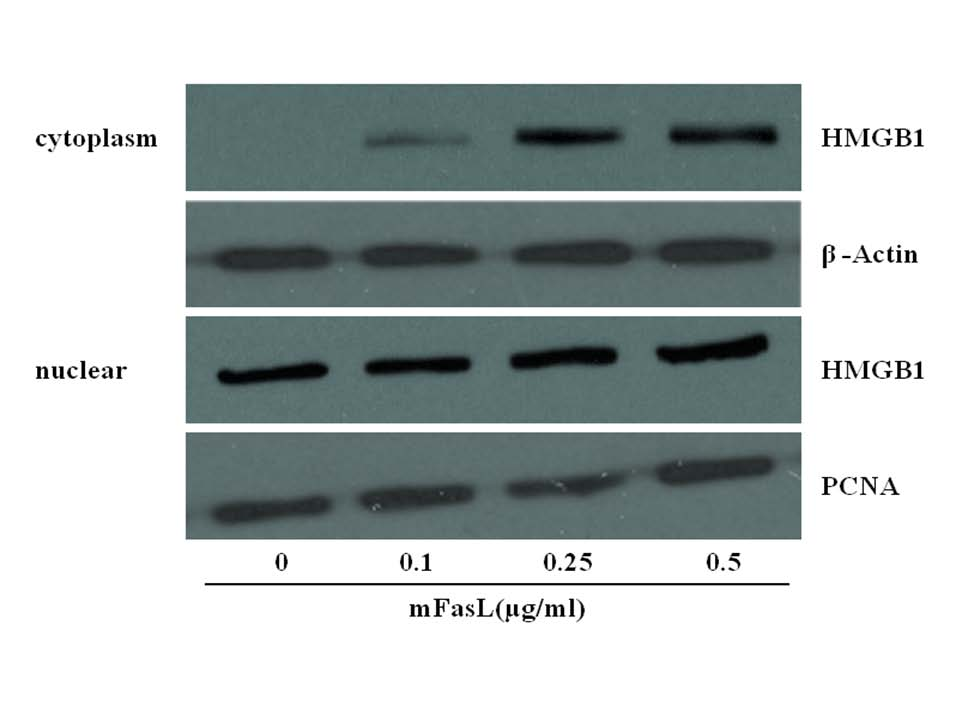
**Fas activation induces cytoplamsic translocation of HMGB1 in primary murine peritoneal mϕ**. Primary murine peritoneal mϕ were stimulated with recombinant mFasL (0 - 0.5 μg/ml) for 2 hr. Cytoplasmic and nuclear extracts were prepared as described in Materials and Methods, and HMGB1 content was determined by immunoblot. Equal loading of samples was confirmed by concomitant immunoblot analysis of the respective fractions using antibodies specific for a nuclear (PCNA) or cytoplasmic (β-actin) protein. Blots are representative of 3 independent experiments with similar results.

HMGB1 is a nuclear DNA-binding protein that plays a functional role in cellular proliferation. In quiescent mϕ, HMGB1 is confined almost exclusively to the nucleus [16,17]. However, HMGB1 can be translocated to the cytoplasm of mϕ following stimulation by specific activating stimuli, such as LPS [29]. To determine if Fas induces HMGB1 cytoplasmic translocation, murine peritoneal mϕ were collected following incubation with mFasL (0 - 0.5 μl/ml) for 2 hr. Cytoplasmic and nuclear extracts were prepared and assyed by specific immunoblot for HMGB1, PCNA (a nuclear protein), and β-actin (a cytoplasmic protein). In quiescent mϕ, HMGB1 was present in the nucleus but undetectable in the cytoplasm. HMGB1 increased dramatically in mϕ cytoplasmic extracts following stimulation with mFasL for 2 hr (Figure 3). These data suggest that Fas induces nuclear-cytoplasmic translocation of HMGB1 in mϕ.

### Release of HMGB1 contributes to Fas-induced pro-inflammatory cytokine production by mϕ

**Figure 4:**
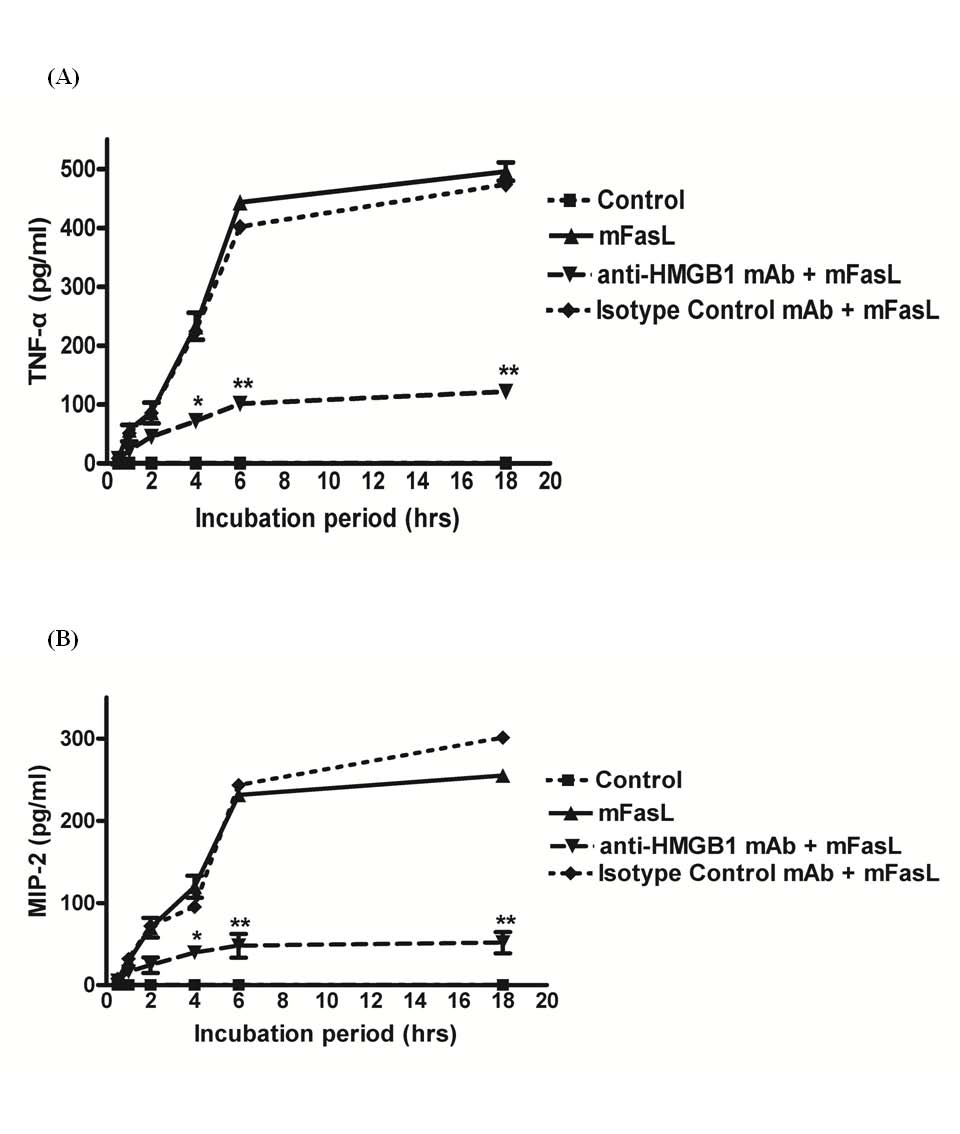
**Neutralization of HMGB1 decreases Fas-induced pro-inflammatory cytokine production by mϕ**. Primary murine peritoneal mϕ were pretreated with anti-HMGB1 mAb (0.25 μg/ml) or isotype control IgG2b (0.25 μg/ml) for 1 hr, prior to stimulation with mFasL (0.25 μg/ml) for the times indicated (0 - 18 hr) as described in Materials and Methods. Pro-inflammatory cytokine production was determined by measurement of (A) TNF-α (4A) and (B) MIP-2 (4B) in culture supernatants by cytokine-specific ELISAs as described in Materials and Methods. Data are presented as mean ± SD of 3 independent experiments, n = 3/group. * *P *≤ 0.05; **P ≤ 0.01.

Both Fas and HMGB1 have previously been shown to induce pro-inflammatory cytokine production by mϕ[10-14,19]. To determine whether HMGB1 contributes to Fas-induced pro-inflammatory cytokine responses, mFasL-induced production of TNF-α (Figure 4A) and MIP-2 (Figure 4B) by wild-type primary murine peritoneal mϕ was measured following 1 hr pre-treatment with either anti-HMGB1 neutralizing mAb (0.25 μg/ml) or an isotype control IgG2b mAb. As expected, the levels of TNF-α and MIP-2 increased in the culture supernatant following mFasL stimulation. Fas-induced production of TNF-α and MIP-2 was significantly decreased by pre-treatment with inhibitory anti-HMGB1 mAb. These results indicate that Fas-mediated release of HMGB1 from mϕ contributes significantly to Fas-induced pro-inflammatory cytokine production by mϕ.

### Fas induced production of pro-inflammatory cytokines is TLR4-dependent

**Figure 5:**
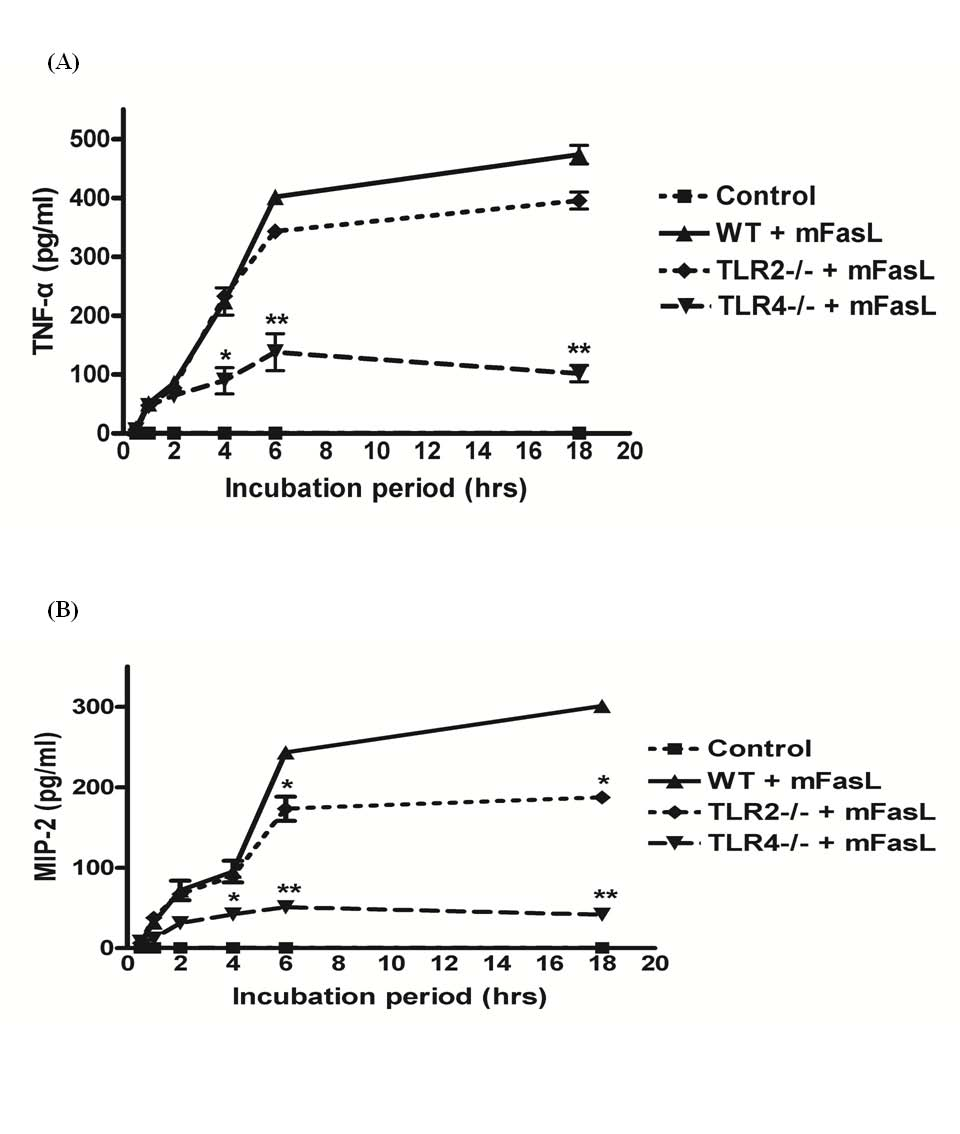
**Fas-induced pro-inflammatory cytokine production by mϕ is TLR4-dependent**. Primary murine peritoneal mϕ from wild-type, *Tlr2*^-/-^, and *Tlr4*^*-/- *^mice were treated with mFasL (0.25 μg/ml) for the times indicated (0 - 18 hr). Pro-inflammatory cytokine production was determined by measurement of (A) TNF-α and (B) MIP-2 in culture supernatants by cytokine-specific ELISAs as described in Materials and Methods. Data are presented as mean ± SD of 3 independent experiments, n = 3/group. * *P *≤ 0.05; **P ≤ 0.01.

To investigate the respective roles of TLR2 and TLR4 on Fas-induced pro-inflammatory cytokine responses, production of TNF-α (Figure 5A) and MIP-2 (Figure 5B) was measured from primary wild-type, *Tlr2*^-/-^, and *Tlr4*^*-/- *^murine peritoneal mϕ incubated with mFasL for 0.5 - 18 hr. TNF-α and MIP-2 production was slightly reduced in wild-type *vs*. *Tlr2*^*-/- *^mϕ. In contrast, the production of both TNF-α and MIP-2 by *Tlr4*^*-/- *^mϕ was strongly decreased in comparison to wild-type mϕ. These results demonstrate that Fas induces pro-inflammatory cytokine production by mϕ via a TLR4-predominate mechanism, but do not entirely exclude a minor contribution from a TLR2-dependent mechanism.

### Fas induces HMGB1 release from mϕ via a TLR4/IRAK4-dependent mechanism

**Figure 6:**
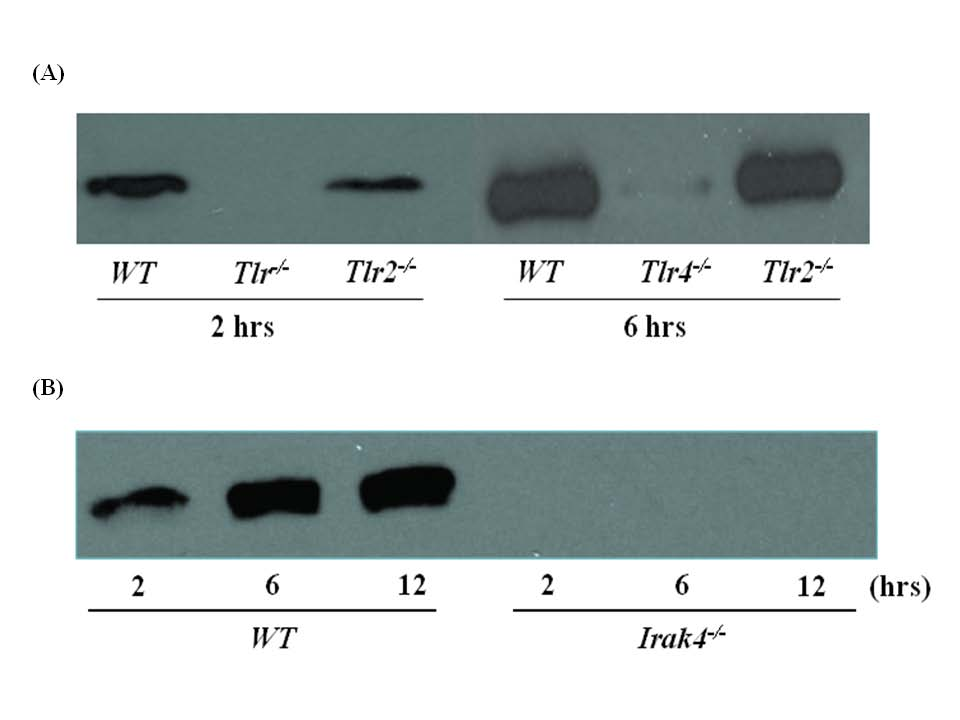
**Fas-induced release of HMGB1 by primary murine peritoneal mϕ is TLR4/IRAK4-dependent**. Primary murine peritoneal mϕ derived from (A) wild-type vs *Tlr2*^*-/- *^vs *Tlr4*^*-/- *^mice, or (B) wild-type vs *Irak4*^*-/- *^mice, were stimulated with mFasL (0.25 μg/ml) for the times indicated (0 - 6 hr) as described in Materials and Methods. HMGB1 release into the concentrated cell culture-conditioned medium was detected by immunoblot as described in Materials and Methods. Results are representative of 3 independent experiments that yielded similar results.

Primary peritoneal murine mϕ from wild-type, *Tlr2*^-/-^, *Tlr4*^-/-^, and *Irak4*^*-/- *^mice were used to investigate if Fas-FasL induced HMGB1 release occurs via a TLR/IRAK4-dependent mechanism. HMGB1 was detected by immunoblot in the culture medium of wild-type and *Tlr2*^-/-^, but not *Tlr4*^-/-^, mϕ following incubation with mFasL (Figure 6A). In addition, Fas stimulation failed to induce HMGB1 release from *Irak4*^*-/- *^mϕ (Figure 6B). These results indicate that Fas induces HMGB1 release from mϕ via a TLR4/IRAK4-dependent mechanism.

## Discussion

HMGB1 has been reported to be actively released by mϕ in response to stimulation with LPS or hydrogen [29,35]. Our results demonstrate that activation of Fas induces not only active but also rapid release of HMGB1 from viable mϕ via a TLR4/IRAK4-dependent mechanism. Furthermore, the data from this study suggest that released HMGB1 functions in an autocrine and/or paracrine manner to mediate Fas-induced pro-inflammatory production by mϕ. To our knowledge, these data are the first to implicate HMGB1 in Fas-mediated physiological responses. These insights significantly advance our understanding of physiological roles of both the Fas/FasL system and HMGB1 in activation of host innate immune responses.

Although generally recognized for its role in mediating caspase-dependent, apoptotic programmed cell death [1-3], the Fas/FasL system is increasingly recognized for its ability to induce pro-inflammatory cytokine production by mϕ via MyD88-dependent, caspase-independent mechanisms [13,14,36,37]. A previous study demonstrated that Fas ligation enhances IL-R1/TLR4 signaling to promote macrophage-mediated inflammation [36]. Specifically, interruption of Fas-FasL signaling was shown to suppress both NF-κB activation and cytokine expression induced by IL-1 and LPS in primary human and murine mϕ [36]. Furthermore, it was suggested that this cross-talk between Fas and IL-R1/TLR4 was mediated by interaction of Fas-associated death domain (FADD) with MyD88. The biological importance of this cross-talk was supported by another study in which sustained functional deficiency of the Fas/FasL system in mice attenuated sustained LPS-induced lung inflammation [38].

The release of HMGB1 into the extracellular environment, where it can function as an endogenous danger signal or 'alarmin' to promote inflammation has been implicated in a number of diseases associated with tissue injury, including sepsis and acute lung injury [28,29]. In the current study, we demonstrated that activation of Fas induced rapid (within 1 hr) release of HMGB1 from RAW264.7 (a murine macrophage cell line) and primary murine peritoneal mϕ (Figure 1). Similar rapid, active release HMGB1 has also been reported in experimental brain ischemia [39]. Following activation of Fas, HMGB1 was detected in the cytoplasm of mϕ, consistent with a process of nuclear-cytoplasmic translocation as previously reported for LPS stimulation [40]. Following this translocation event, HMGB1 was subsequently detected in the extracellular milieu of mϕ (Figure 3). Importantly, Fas-induced HMGB1 release was not associated with cell death (Figure 2) and was remarkably more rapid than HMGB1 release induced by LPS (Figure 1), a known stimulus for HMGB1 release from mϕ [32,40].

Surprisingly, experiments performed employing murine mϕ with targeted genetic deletions of *Tlr2, Tlr4, *and *Irak4 *demonstrated that the Fas/FasL system induced HMGB1 release primarily via a TLR4/IRAK4-dependent mechanism (Figure 6). Interestingly, previous studies have demonstrated TLR4-mediated activation of HMGB1 translocation and subsequent release of HMGB1 from distinct cell types, including mϕ [29,40-43]. Taken together, our data are consistent with a proposed 3-step model for Fas-mediated activation of pro-inflammatory cytokine production by mϕ: 1) Fas-induced nuclear-cytoplasmic translocation of HMGB1; 2) subsequent release of cytoplasmic HMGB1 by Fas-activated mϕ; and 3) autocine/paracrine-mediated feedback by HMGB1 to activate TLR4 and promote TLR4/IRAK4-dependent pro-inflammatory cytokine production. Based on our observation that mϕ viability was not affected by Fas activation, we believe that Fas-mediated HMGB1 release occurs via the previously described active pathway for HMGB1 release from viable cells [29,40-43]. This pathway appears to be calcium/calmodulin-dependent and involve HMGB1 hyperacetylation and/or phosphorylation [28,40-43]. Overall, our findings are consistent with previous reports that the TLR4/MyD88/IRAK4 complex not only serves to detect and initiate intracellular signaling in response to exogenous pathogens but also functions to secrete HMGB1 from mϕ into the extracellular environment [27-29,40-43].

Our current study strongly supports a role for HMGB1, acting as an autocrine and/or paracrine mediator, in Fas-induced pro-inflammatory cytokine production by mϕ. Neutralization of HMGB1 using an inhibitory anti-HMGB1 mAb significantly decreased Fas-induced production of TNF-α and MIP-2 by primary murine peritoneal mϕ (Figure 4). The observation that Fas-induced production of TNF-α and MIP-2 was markedly decreased in *Tlr4*^*-/- *^mϕ vs wild-type mϕ (Figure 5) is consistent with previous reports that HMGB1 may function predominately as a TLR4 agonist to promote macrophage-mediated inflammation [26,28,30,44,45]. However, our results do not exclude the possibility that TLR2 and/or RAGE may serve as receptors for HMGB1 on mϕ [25-27,44].

Previous studies have identified several mechanisms for Fas-induced pro-inflammatory responses in mϕ. Miwa et al. reported that Fas induced release of IL-1β from mϕ via a caspase 1-independent pathway to promote inflammation [8]. Ma et al. extended our understanding of Fas-mediated inflammation by demonstrating cross-talk in mϕ, whereby Fas enhanced signaling via either the IL-1R1 and/or TLR4 pathway to promote chronic inflammation [36]. Previous studies from our group demonstrated that Fas could activate pro-inflammatory cytokine production by mϕ via a MyD88-dependent, caspase-independent pathway [14]. Our current study has identified a unique mechanism for Fas-mediated inflammation, in which HMGB1 is rapidly released by viable mϕ via a TLR4/IRAK4-dependent mechanism and functions in an autocrine and/or paracrine manner to stimulate pro-inflammatory cytokine production.

In summary, this study demonstrates a previously undescribed mechanism for Fas-mediated inflammation involving rapid, TLR4/IRAK4-dependent release of pro-inflammatory HMGB1 from mϕ. Furthermore, our observations implicate novel physiological cross-talk between Fas, TLR4, and HMGB1 in the pathogenesis of mϕ-mediated inflammation. These results suggest the potential utility of therapeutic interventions directed against HMGB1 in pathophysiological disease states associated with mϕ-mediated inflammation and tissue injury.

## Competing interests

The authors declare that they have no competing interests.

## Authors' contributions

FW and WCL designed the study, performed the experiments, interpreted the data and drafted the manuscript. ZYL performed animal experiments, and HY provided essential reagents for conducting the experiments involving neutralization of HMGB1. MH and KCK assisted in study design and interpretation of data. All authors read and approved the final manuscript.

## About the authors

W. Conrad Liles, M.D., Ph.D. Professor and Vice-Chair of Medicine; Director, Division of Infectious Diseases; Senior Scientist, McLaughlin Centre for Molecular Medicine; Senior Scientist, McLaughlin-Rotman Centre for Global Health; Canada Research Chair in Infectious Diseases and Inflammation; University of Toronto

WCL received his undergraduate education at Williams College (Williamstown, MA, USA), then entered the National Institutes of Health (NIH)-sponsored MD-PhD program (Medical Scientist Training Program) at the University of Washington and graduated in 1987 with an MD and PhD in pharmacology. Following residency in Internal Medicine at Massachusetts General Hospital from 1987-1990, he returned to the University of Washington where he served as Chief Medical Resident in 1991 and as a Fellow in Infectious Diseases from 1992-1995. In 1996, he was named to the faculty at the University of Washington as Assistant Professor of Medicine in the Division of Allergy and Infectious Diseases and rose to the rank of Professor of Medicine and Adjunct Professor of Pathology. In March 2006, WCL moved to the University of Toronto to assume the positions of Vice-Chair of Medicine and Director, Division of Infectious Diseases. He was attracted to the University of Toronto by the opportunities to build translational research programs in sepsis, inflammation, emerging infectious diseases of public health importance. He is the recipient of a Canada Research Chair in Inflammation and Infectious Diseases and a member of the McLaughlin Centre for Molecular Medicine, the McLaughlin-Rotman Centre for Global Health, and the Toronto General Research Institute. As author of more than 135 peer-reviewed manuscripts and 35 book chapters, WCL maintains an active translational research program in host defense, inflammation, innate immunity, sepsis, immunodeficiency disorders, and immunomodulatory therapy, while serving as a chartered member of the Immunity and Host Defense Study Section of the NIH. In 2004, WCL received the Outstanding Investigator Award from the Western Society for Clinical Investigation (WSCI). He has been elected to Fellowship in the American College of Physicians (ACP) and the Infectious Diseases Society of America (IDSA).
